# Interesting Response with the Delivery of Right Ventricular Extrastimulus of Increasing Prematurity

**DOI:** 10.19102/icrm.2021.120706

**Published:** 2021-07-15

**Authors:** Krishna Kumar Mohanan Nair, Narayanan Namboodiri, Ajitkumar Valaparambil

**Affiliations:** ^1^Department of Cardiology, Sree Chitra Tirunal Institute for Medical Sciences and Technology, Thiruvananthapuram, Kerala, India

**Keywords:** Retrograde conduction, right bundle branch block, ventricular extrastimulus

## Abstract

A 45-year-old man with no structural heart disease underwent an electrophysiology study for recurrent episodes of palpitation. There was no evidence of pre-excitation on the baseline electrocardiogram. Baseline intervals, including the A–H and H–V intervals, were within the normal limits and remained so during the electrophysiology study. An interesting response was observed with the delivery of ventricular extrastimuli with increasing prematurity from the right ventricular apex, triggering consideration of the possible mechanism.

A 45-year-old man with no structural heart disease underwent an electrophysiology study for recurrent episodes of palpitation. There was no evidence of pre-excitation on the baseline electrocardiogram (ECG) and baseline intervals, including the A–H and H–V intervals, were within the normal limits. During the electrophysiology study, these intervals remained within normal limits. However, ventricular extrastimuli (VES) delivered with increasing prematurity from the right ventricular (RV) apex led to an interesting response.

**Figure 1A** shows VES delivered after a drive train of eight beats with a pacing cycle length of 450 ms at a prematurity of 280 ms. In response, the V–H and V–A intervals measured 60 ms and 105 ms, respectively. Meanwhile, with a VES of 260 ms, the V–H and V–A intervals increased to 110 ms and 155 ms, respectively **(Figure 1B)**. The atrial activation pattern remained concentric with both coupling intervals, and the H–A intervals were 45 ms with both coupling intervals.

## Discussion

An increase in the V–A interval following an increase in the prematurity of the VES may be (1) due to a sudden shift in retrograde conduction from a fast atrioventricular (AV) nodal pathway to a slow AV nodal pathway, (2) the result of accessory pathway conduction, or (3) a consequence of retrograde right bundle branch block (RBBB). With the development of retrograde RBBB, retrograde conduction occurs via transseptal activation and retrograde conduction through the left bundle in order to reach the His bundle. This is suggested by an increase in the V–H interval of at least 50 ms in response to VES or a V–H jump.^1^

In the index case, in response to an increase in the prematurity of the VES by 20 ms, an increase in the V–A interval by 50 ms was preceded by the V–H jump. This suggests not only the development of retrograde RBBB but also retrograde nodal conduction as the increments in the V–H interval and the V–A interval were identical to one another. With exclusive retrograde nodal conduction, the His bundle will be activated invariably prior to the AV node and, hence, the development of retrograde RBBB will prolong the V–A interval. The increment in the V–A interval will be at least as much as the increase in the V–H interval. When the retrograde conduction is extranodal or via the accessory pathway, despite the development of retrograde RBBB, there will be no change in the V–A interval.

The trace shows the development of retrograde RBBB development followed by retrograde nodal conduction in response to VES of increasing prematurity.

## Figures and Tables

**Figure 1:**
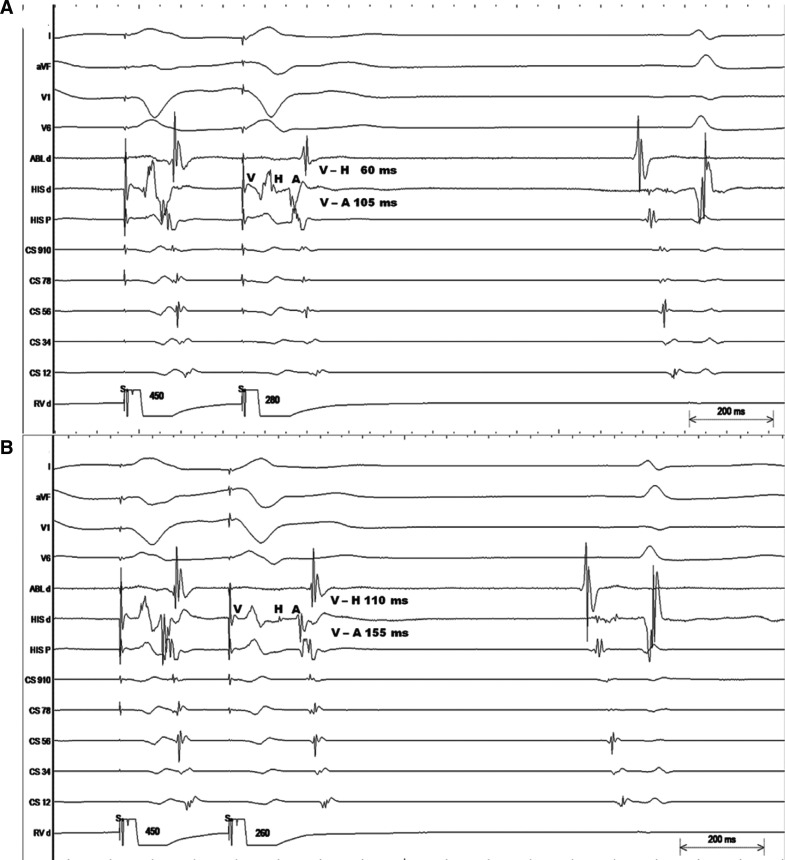
**A:** Represents surface ECG (I, aVF, V1, V6) and intracardiac electrograms at mapping and ablation catheter distal at the right atrial appendage (ABL d), His-bundle electrogram distal (HIS d), His-bundle electrogram proximal (HIS p), coronary sinus (CS) 9,10 dipoles at CS ostium, CS 1,2 dipoles at distal CS, and RV apex distal (RV d) showing a response to VES delivered at a prematurity of 280 ms. **B:** Surface ECG (I, aVF, V1, V6) and intracardiac electrograms at mapping and ablation catheter distal at the right atrial appendage (ABL d), His bundle electrogram distal (HIS d), His bundle electrogram proximal (HIS p), CS 9,10 dipoles at CS ostium, CS 1,2 dipoles at distal CS, and RV apex distal (RV d) showing response to VES delivered at a prematurity of 260 ms.
